# Aerial Imagery Based on Commercial Flights as Remote Sensing Platform

**DOI:** 10.3390/s20061658

**Published:** 2020-03-17

**Authors:** Toni Mastelic, Josip Lorincz, Ivan Ivandic, Matea Boban

**Affiliations:** 1 ETK Research, Ericsson Nikola Tesla d.d., Poljicka cesta 39, Split 21000, Croatia; 2 Faculty of Electrical Engineering, Mechanical Engineering and Naval Architecture (FESB), University of Split, R. Boskovica 32, Split 21000, Croatia; josip.lorincz@fesb.hr (J.L.); ivan.ivandic.00@fesb.hr (I.I.); matea.boban.02@fesb.hr (M.B.)

**Keywords:** remote sensing, aerial imagery, commercial flights, land coverage, temporal, spatial

## Abstract

Remote sensing is commonly performed via airborne platforms such as satellites, specialized aircraft, and unmanned aerial systems (UASs), which perform airborne photography using mounted cameras. However, they are limited by their coverage (UASs), irregular flyover frequency (aircraft), and/or low spatial resolution (satellites) due to their high altitude. In this paper, we examine the utilization of commercial flights as an airborne platform for remote sensing. Namely, we simulate a situation where all aircraft on commercial flights are equipped with a mounted camera used for airborne photography. The simulation is used to estimate coverage, the temporal and spatial resolution of aerial imagery acquired this way, as well as the storage capacity required for storing all imagery data. The results show that Europe is 83.28 percent covered with an average of one aerial photography every half an hour and a ground sampling distance of 0.96 meters per pixel. Capturing such imagery results in 20 million images or four petabytes of image data per day. More detailed results are given in the paper for separate countries/territories in Europe, individual commercial airlines and alliances, as well as three different cameras.

## 1. Introduction

Remote sensing includes many applications such as [1]: planning towards sustainable agriculture, monitoring and studying the bio-geological characteristics of oceans, water security and management, environmental assessment and monitoring, disaster monitoring and mitigation, weather and climate studies, and infrastructure development. All of these applications utilize aerial imagery as a remote sensing approach in the form of raster geo-referenced images containing various spectral bands defined by their wavelength and bandwidth. However, while bands depend on the features of a mounted camera, the frequency acquisition of those images, coverage, and partly their resolution depend on the object to which the camera is mounted [2].

On the one hand, due to their high altitude, satellites provide wide coverage at the expense of image resolution, while unmanned aerial systems (UAS) excel in resolution, but lack in coverage [3]. Moreover, imagery taken from an aircraft provides optimal resolution and coverage. On the other hand, both UAS and aircraft provide ad hoc frequency for aerial imagery due to their inability to remain in the air for a long time, unlike satellites that orbit the Earth in regular intervals [2]. However, these regular intervals usually count in days or hours, e.g., Sentinel satellites provide images every 4.5 days [4], which is insufficient for modern applications that go towards (near) real-time analysis [5]. Geostationary satellites compensate this by remaining over a single location; however, their coverage is limited by an extremely narrow ring in the plane of the Equator and lacks high resolution due to their high altitude [5].

In this paper, we examine the feasibility of remote sensing by means of commercial flights, where an aircraft is mounted with a camera and thus used as an airborne platform. A dataset from Flightradar24 (www.flightradar24.com) is taken containing 32,459 flights over Europe in a single day. Flight trajectories are interpolated and used for estimating the coverage, temporal, and spatial resolution of aerial images, as well as their storage size, as if they were taken from those flights. The simulation is performed by taking parameters for three different cameras, separately summarizing data by airline companies and alliances, as well as by presenting results for all of Europe and each European country/territory. The simulation results show that Europe is 83.28 percent covered with aerial photography every half an hour with an average spatial resolution expressed as a ground sampling distance (GSD) of 0.96 meters per pixel. Such an airborne platform produces 20 million images or 5 petabytes of image data per day.

More detailed results show that the Star Alliance alone covers 57.80 percent of Europe with 5.56 daily flyovers on average over a certain area, which results in over 500 terabytes of data. By looking at individual airline companies, Ryanair alone covers 34 percent of Europe. Combined with Turkish Airlines, they cover half of Europe. Moreover, half of Europe is covered by at least 10 airlines and by at least one flight every hour. The most flyovers per day are achieved for Turkey and Germany with 1306 and 1094 flyovers, respectively. However, the highest average number of flyovers is given for Luxembourg with 224 flyovers per day. Finally, the best spatial resolution is achieved for smaller countries/territories in Europe such as the Vatican, Gibraltar, and Monaco, while France, Germany, and Spain require the most storage capacity.

According to our knowledge, this is the first paper that analyses the applicability of airline operated commercial aircraft for transporting passengers and cargo as a platform for aerial imagery. The goal of the paper is to answer the question related to the applicability of exploiting commercial flights as an airborne platform for performing aerial imagery in the context of coverage, the temporal and spatial resolution of aerial images, as well as their storage size.

The rest of the paper is structured as follows. Section 2 gives an overview of aerial imagery and relevant work. The methodology used in this work is explained in Section 3 with results presented in Section 4. Finally, a discussion and conclusion with future work are given in Section 5 and Section 6, respectively.

## 2. Background

The term remote sensing is used to describe information gathering from a distant target, i.e., an object or a phenomenon, without making physical contact with the target [2]. It is typically done by using satellites, aircraft, and recently UAS [6], which collect data reflected from the Earth’s surface. The information is acquired through different signals such as electromagnetic radiation, force fields, or acoustic energy, where the signal is either created by the sensor (active sensors) or is found in nature as-is (passive sensors) [7]. Active sensors send a signal towards the target and then read it as it reflects from the target, while passive sensors collect data that are naturally reflected by the target. That being said, an advance of satellite and UAS technologies has made aerial imagery a relevant source of passive sensor data for remote sensing [3].

Aerial imagery, also known as airborne photography, is based on taking images of the Earth’s surface from a flying object(s). Since the first airborne photography taken by G. F. Tournachon in 1858 from a hot air balloon just outside Paris, flying objects as platforms used for aerial imagery have radically changed [6,8]. During the last more than 160 years, besides hot air balloons, platforms used for aerial imagery include blimps, kites, fixed-wing manned aircraft, rockets, pigeons, parachutes, helicopters, unmanned aerial vehicles (UAVs or “drones”), and satellites [6].

Among all mentioned flying platforms used for aerial imagery, manned aircraft historically played an important role and are still seen as a classical sensing platform for aerial imagery. The first known airborne photography was taken from the airplane over Le Mans, France. by L. P. Bonvillain in 1908 with the airplane inventor W. Wright as a pilot [9]. Massive usage of aerial imagery taken from cameras installed on fixed-wing manned aircraft started in World War One (WW-I) for the purpose of reconnaissance missions, which resulted in up to then thousands of images per day taken during the middle of the WW-I. By the beginning of the Second World War (WW-II), the interpretation of aerial imagery taken from the aircraft started to be used in many different fields and applications such as agriculture, archeology, forestry, glaciology, etc. [10]. Vast usage of aerial imagery taken from manned aircraft continued during WW-II. Besides military reconnaissance purposes used on all sides during WW-II, aerial photography taken from the aircraft entered in the media such as newspapers, magazines, and movies [6,8].

After WW-II, manned aircraft become the most often used means for ensuring the platform for aerial imagery, which enabled different professions and industries to have more powerful options in photographing, measuring, surveying, and mapping different spaces and places. For instance, the usage of cameras placed on fixed-wing manned aircraft enabled the development of different disciplines such as photogrammetry, which provides object’s geometric information derived with the help of image measurements [10]. Moreover, remote sensing with the use of aerial survey cameras mounted on fixed-wing manned aircraft significantly contributed to the development of Geographic Information Systems (GIS), which are based on generating huge volumes of digital images needed for geocoded rectification and analysis [10,11]. Besides historical usage of aircraft as a platform for aerial photo or video imagery, the latest advancements in areal remote sensing ensure that aircraft become one of the most important platforms for caring airborne laser imaging detection and ranging (LiDAR) systems [10]. Such systems enable automated acquisition of three-dimensional (3D) representation of objects by measuring the reflected laser light to which objects are exposed. Additionally, aircraft as the airborne platform are used for hosting sensing equipment, which works in multispectral, hyperspectral, and microwave frequency bands [12].

According to the forecasts, the global remote sensing service market was estimated at USD 9.70 billion in 2016, and with a projected compound annual gross rate (CAGR) of 15.14% from 2017 to 2022, it will reach USD 21.62 billion by 2022 [13]. Although the remote sensing service market based on manned aircraft is comprised of many segments and applications, the following main segments currently dominate the market: precision farming, coastal analysis, mineral exploration mapping, disaster management, pipeline monitoring, defense, and intelligence [13]. As an important part of the global remote sensing market, the global aerial imaging market was estimated at USD 1.439 billion in 2017, where UAS and fixed-wing manned aircraft took almost equal parts of ca. 45% in the overall share of all remote sensing platforms [14]. Forecasts for the period 2018-2025 show an increase of the global aerial imaging market with CAGR (Compound Annual Growth Rate) of 14.2%, which results in the global aerial imaging market reaching USD 4.125 billion by 2025.

Despite estimations of the forecast domination by the usage of UASs, and more specifically small UAS (sUAS) [3], as a platform in global remote sensing by 2024 (with aerial imaging market share of 78% in comparison with other remote sensing platforms) [15], fixed-wing manned aircraft will continue to have an important role as a platform for remote sensing in the form of aerial imagery. The reason can be found in the essence of fixed-wing aircraft as a sensing platform, which can be used for quick and frequent log-range and vast area remote sensing (aerial imagery) [16]. This explains why the exploitation of fixed-wing aircraft as a remote sensing platform will remain irreplaceable for many specific sensing purposes and applications.

Conventional fixed-wing aircraft currently used for remote sensing vary in size from small single-engine piston-powered airplanes to sophisticated multi-engine propeller or jet airplanes [17]. The selection of the aircraft for aerial imagery is generally based on the meteorology and geography of the surveying area, the operating and capital costs, the operators’ intended use, performance, safety, maneuverability, and ease of the airplane adaptation to the remote-sensing operations [18].

Fixed-wing aircraft used for remote sensing are usually based on adapted light aircraft or military types of aircraft. Aircraft adaptations can be expensive, and it is generally more economical to select an aircraft that requires minimum adaptations. For that reason, this paper analyses the possibility of performing aerial imagery with commercial fixed-wing manned airliners during their regular flights as the airborne platform.

## 3. Methodology

The characteristics of aerial imagery are defined by four resolution types, namely spatial, spectral, temporal, and radiometric resolution [2,3].

- Spatial resolution is defined as the smallest object that can be resolved from the remote sensing image. It is commonly measured as the ground sampling distance (GSD), which defines a distance between the centers of two pixels, i.e., a ground sampling distance of 10 meters means that every pixel of an image obtained by the sensor covers a ground area of 10 m × 10 m.
- Temporal resolution represents the frequency at which a sensor reads a new value, e.g., a temporal resolution of 5 days means that the sensor takes a new aerial image every 5 days.
- Spectral resolution refers to the number of spectral bands a remote sensor is capable of reading, e.g., basic remote sensors can detect only visible light in red, green, and blue bands, while advanced ones can read multiple narrow bands in the higher and lower spectrum, such as near-infrared.
- Radiometric resolution is the ability of a remote sensor to distinguish small differences in electromagnetic energy, e.g., within a single band, a sensor can distinguish 8 bits of data, which equals 256 different values, which is then stored as a value of a single-pixel in an aerial image.

In this paper, we focus on spatial and temporal resolutions, as these characteristics are affected by an airborne platform, namely its flyover frequency and altitude. Spectral and radiometric resolutions are direct products of the used camera and are thus out of the scope of this paper. However, since spatial resolution is also affected by the used camera, we include it in our analysis by applying the characteristics of three different cameras in our simulations and compare the results. In order to estimate coverage, the temporal and spatial resolutions of aerial images, along with their data size as if they were acquired from commercial aircraft during their regular flights, five steps are required:

1. The dataset containing positions and altitudes of commercial flights over a certain area, as well as some additional metadata information for more comprehensive analysis.
2. Interpolation of the dataset containing flight trajectories, as well as polygons representing the viewpoint of a camera mounted on an aircraft taking into consideration flight trajectory, altitude, as well as camera characteristics.
3. Projection of the land mass and trajectories onto the map, which is required for calculating area size, coverage, and other statistics.
4. Account for the overlapping of images to follow a common practice in airborne photography where images are taken successively to cover a certain area with more than one image.
5. Data clustering and analysis based on polygon intersections between flights themselves, as well as individual countries. Moreover, additional data clustering is done based on flight metadata, such as individual airlines or alliances.

All five steps are described in the following subsections, namely Section 3.1, Section 3.2, Section 3.3, Section 3.4 and Section 3.5.

### 3.1. Flight Dataset

In this work, we utilized data contributed by Flightradar24 AB, which provides a professional data service of historic flight position data based on the recorded positions of live aircraft. Data were acquired from a network of receivers that capture ADS-B (Automatic Dependent Surveillance-Broadcast) or mode-S (Selective) transponder signals from aircraft. Transponder data were then combined with Flightradar24’s reference database in order to get a complete dataset containing all relevant information. The resulting dataset was comprised of two segments, namely flight data and trajectory data, as depicted in Table 1 and Table 2, respectively.

We used the call sign from Table 1 to extract the ICAO (International Civil Aviation Organization) three letter airline code and map it to the airline name, as well as an alliance to which the airline belongs. The equipment and aircraft ID were used to filter out airport ground vehicles and private aircraft, while the flight number and call sign were used to identify commercial flights. The flight ID was used to identify flights uniquely and map trajectory data to them from Table 2.

Aircraft positions were updated every 5 seconds during take-off and landing due to a rapid change in direction, and updating was increased to a maximum of 60 seconds during steady flight. Moreover, the availability of the aircraft positional data was strictly dependent on the transponder broadcast and the coverage of a nearby receiver in the geographical region in which the aircraft was flying. Consequently, estimated flight positions were excluded for areas with no direct coverage available, as well as for those that had their information restricted or blocked.

**Figure 1:**
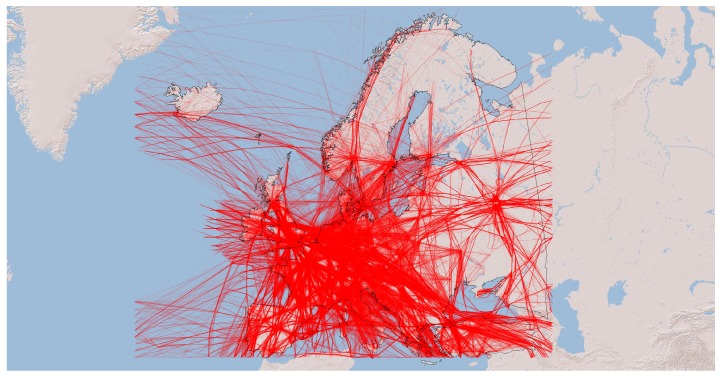
Visualization of all flight trajectories from the dataset (map tiles by ESRI, ArcGIS licensed under the ESRI Master License Agreement).

To summarize, the dataset used in this research contained 47,126 trajectories recorded during a single day, namely 31 January 2018 over Europe, out of which 32,459 were identified as commercial flights (passenger or cargo). Others included airport ground vehicles, private aircraft, flights without a call sign, UFOs (i.e., unidentified flying objects, which refer to objects that were not identified as commercial or private aircraft, rather then an extraterrestrial life form or aliens), and grounded flights (i.e., flights that were recorded only taxiing on the ground). Actual commercial flights were done by 363 different companies and 7392 unique aircraft, with a total of 7,007,801 positions and their altitudes, which were used for interpolating flight trajectories. Figure 1 depicts the trajectories of all commercial flights from the described dataset.

### 3.2. Interpolating Flight Trajectories

The aircraft trajectory data described above were used for generating flight trajectories, as well as polygons representing a continuous field of view (FOV) for a moving camera mounted on an aircraft. For this purpose, several cameras were selected, going from entry point cameras to professional cameras commonly used for satellite imagery, for interpolating FOV polygons as if cameras were mounted on the aircraft from the dataset. Table 3 lists the used cameras and their characteristics.

**Figure 2:**
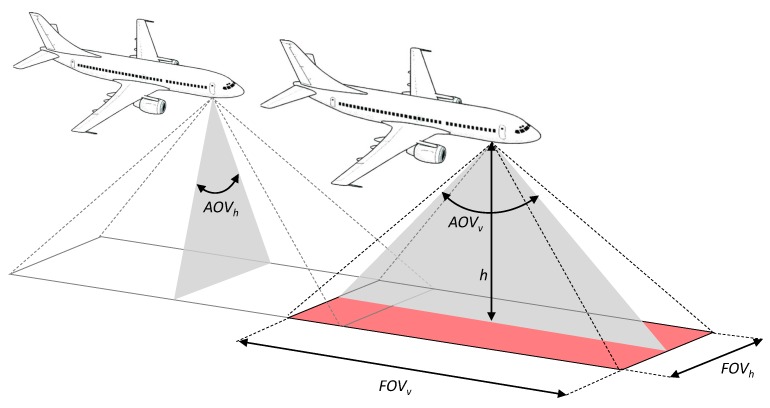
Field of view (FOV ) in relation to the angle of view (AOV) and aircraft altitude *h*.

Firstly, the angle of view (AOV) for each camera was calculated using Equation (1), both for horizontal ($AOV_{h}$) and vertical ($AOV_{v}$) angles depicted in Figure 2, where *s* represents either the width or height of the sensor in millimeters, while *f* stands for the focal length. Table 3 shows the calculated AOV values for all selected cameras.

(1) $$AOV\left[\mathrm{DEGREES}\right]=2\cdot \arctan\left(\frac{s}{2\cdot f}\right)\cdot \left(\frac{180}{\Pi }\right)$$

Secondly, the field of view (FOV) was calculated for each recorded position by taking its altitude *h* and previously calculated AOV, as shown in Equation (2). The obtained value represents either the horizontal ($FOV_{h}$) or vertical ($FOV_{v}$) distance in meters, as depicted in Figure 2. On the one hand, the horizontal distance was used for creating polygons representing continuous FOV for estimating land coverage and the spatial and temporal resolution. On the other hand, vertical distance was used for estimating the number and size of the images acquired during a flight in order to obtain a continuous landscape image.

(2) $$FOV\left[\mathrm{METERS}\right]=2\cdot tan\left(\frac{AOV}{2}\right)\cdot h$$

**Figure 3:**
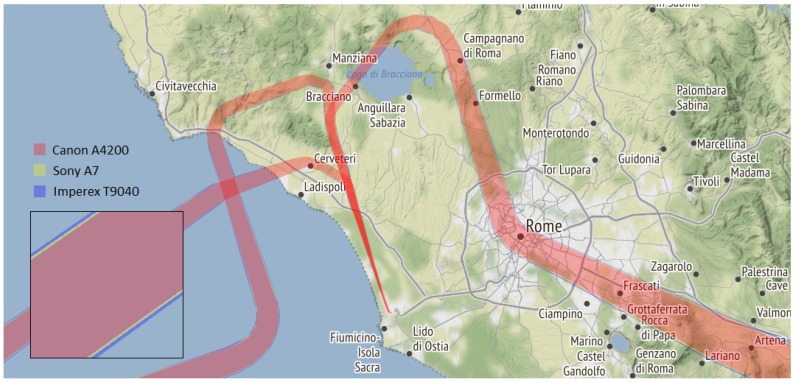
Example of trajectory interpolation and field of view polygons for three aircraft (map tiles by Stamen Design, under CC BY 3.0. Data by OpenStreetMap, under Open Data Commons Open Database License).

Polygons were created for each flight separately, where each polygon represented the FOV of a single aircraft during its flight. For instance, three polygons are depicted in Figure 3 representing three flights, i.e., three aircraft taking off from Rome Fiumicino Airport. Their FOV was very narrow during take-off, while it became broader as the aircraft gained altitude. Additionally, Figure 3 shows three different polygons for each flight representing three different cameras from Table 3. Canon A2400 had the smallest coverage depicted in red, while Sony A7 and Imperex T9040 covered additional areas depicted in yellow and blue, respectively.

### 3.3. Projecting Flights onto a Map

Polygons created in the previous section were represented with geodetic coordinates, i.e., latitudes and longitudes. In order to calculate their area size, they were translated into plane coordinates, which was performed by projecting the polygons onto the map. We used the EPSG:3035 (EPSG Geodetic Parameter Dataset: https://epsg.io/3035) also defined as ETR S89 (European Terrestrial Reference System 1989) Coordinate Reference System (CRS) that targets Europe, along with the Lambert azimuthal equal area (LAEA) [19] map projection commonly used for statistical mapping where true area representation is required. The true or equal area was required when drawing polygons onto the map and calculating statistics from the generated heat map, such as temporal resolution. Otherwise, the calculation would provide disproportional values for northern countries when using Mercator map projection, for instance.

### 3.4. Overlapping the Images

**Figure 4:**
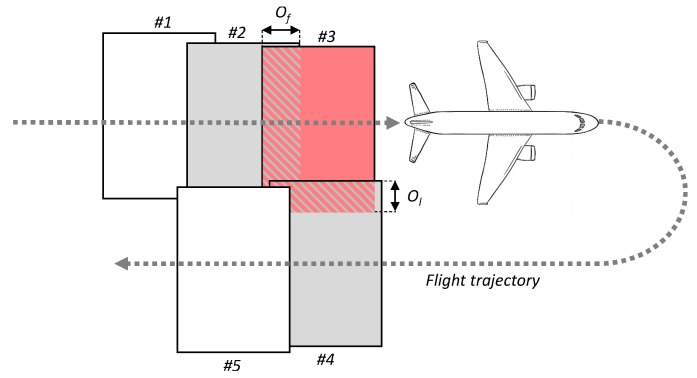
Forward and lateral overlap in aerial imagery.

In order to avoid the loss of data and achieve higher accuracy in image processing, aerial imagery is commonly taken with some redundancy by overlapping the images. An overlap is defined as the amount by which one image includes the area covered by another image, commonly expressed as a percentage. It comprises two types, namely forward overlap and lateral overlap [20]. The former defines an overlap between images along the same flight line as depicted with $O_{f}$ in Figure 4, while the latter defines an overlap between images on adjacent flight lines as depicted with $O_{l}$ in the same figure.

On the one hand, we used forward overlaps in this paper to estimate the number of images taken on each flight. The estimation was used for calculating storage capacity in terabytes required for storing all acquired images. The minimum required forward overlap is 60 percent, as suggested in [20]. On the other hand, lateral overlaps are commonly used during planned flight routes, such as satellite trajectories or drone survey flights. Their common value is between 25 and 30 percent. However, since commercial flights did not follow topographical mapping routes, they were not used in this paper.

### 3.5. Clustering Results Approach

**Figure 5:**
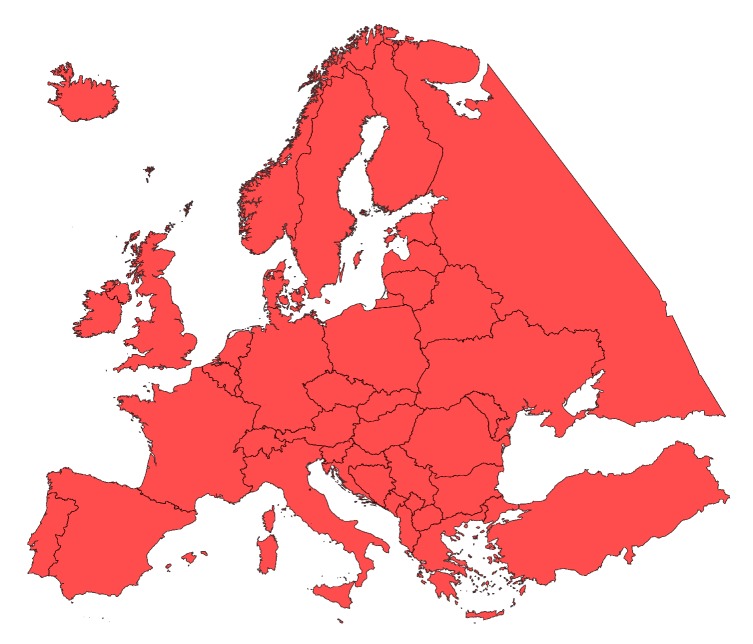
Area covered by the dataset.

All analysis was done over 52 countries and territories listed in Table 4 and depicted in Figure 5. Note that the selection of countries and territories was done based on the coverage of the dataset and the availability of geojson features, which are part of the European land mass. That said, the simulation results represent the entirety of Europe, as well as each country separately where necessary. Furthermore, results for different cameras listed in Table 3 are also presented separately where appropriate.

In order to provide more fine-grained results, the dataset was further clustered by individual airlines and alliances, that is the results for land coverage, temporal and spatial resolution, and storage capacity were given in total, as well as for different clusters of airlines and alliances. As previously mentioned, the dataset contained flights from 363 different airlines, where some of them are members of one of three alliances, namely OneWorld (www.oneworld.com), SkyTeam (www.skyteam.com), and Star Alliance (www.staralliance.com). Table 5 shows some basic statistics of alliances from the dataset.

Due to the variable sampling rate of a transponder depending on the aircraft activity as described in Section 3.1, all averaged statistical values were weighted against the traveled distance. For instance, altitude and speed values in Table 5 represent weighted averages of altitude and speed, respectively. This way, we mitigated biased results of the values that were collected more frequently. The same approach was taken for all other calculations performed in this paper unless otherwise stated.

## 4. Results

In this section, we apply our methodology described in the previous section on the dataset and present the results for four different aspects, namely land coverage, the temporal and spatial resolution, as well as required storage capacity for implementing such an aerial imagery system based on commercial flights as an airborne platform.

### 4.1. Land Coverage

**Figure 6:**
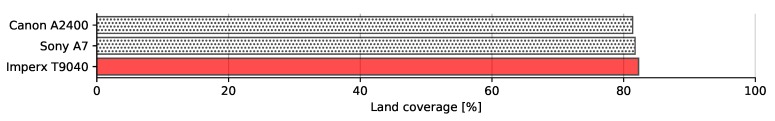
Land coverage of the entirety of Europe by individual cameras.

Land coverage represents an area that is covered by aerial imagery taken from commercial flights as if they were mounted with cameras. Three cameras were used, namely Canon A2400, Sony A7, and Imperx T9040, as an industry camera for aerial imagery, all listed with their characteristics in Table 3. As seen in Figure 6, all cameras exhibited similar land coverage due to their similar FOV values, which determined the area captured by the camera (also seen in Figure 3). Consequently, further analysis for land coverage was done only with Imperx T9040.

**Figure 7:**
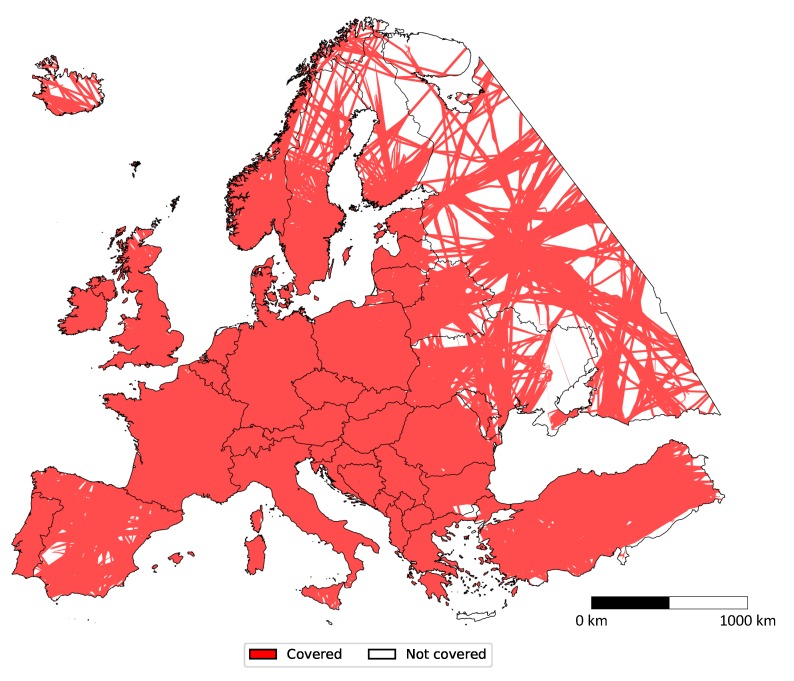
Land coverage of European countries/territories by all flights with Imperx T9040.

**Figure 8:**
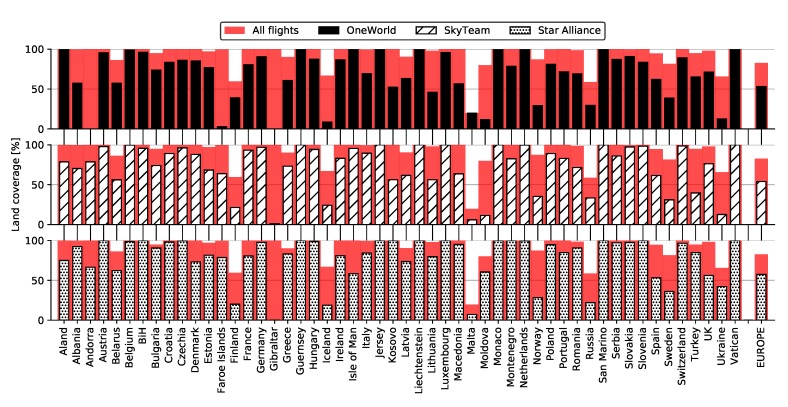
Land coverage by all flights (background red) and individual alliances (monochrome bars) with Imperx T9040.

Figure 7 depicts land coverage by Imperx T9040 over Europe with visible country and territory borders. Western, Central, and Southern Europe are almost entirely covered, while the east and north are scarce. More detailed coverage is depicted in Figure 8 showing percentages for each country separately (background red). Bordering countries such as Spain, Greece, Malta, and Iceland exhibited slightly lower land coverage, while Russia and Ukraine were only 60 percent covered. All other countries were almost one hundred percent covered, while the entirety of Europe was covered by 83.28 percent.

**Figure 9:**
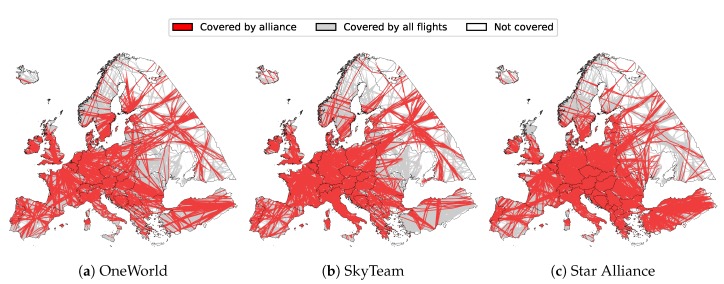
Land coverage by individual alliances with Imperx T9040.

Figure 8 also shows land coverage percentages for individual alliances. Slightly higher coverage was exhibited by Star Alliance (57.80%) for the entirety of Europe, mostly due to its better coverage of east and southeast Europe. This includes countries such as Moldova, Ukraine, Turkey, and Greece, as seen both from the graph, as well as Figure 9, depicting land coverage maps for each alliance separately. OneWorld and SkyTeam covered 53.34% and 54.30%, respectively.

**Figure 10:**
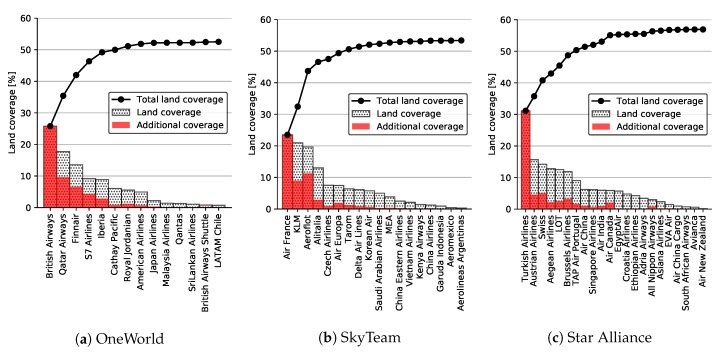
Cumulative land coverage by individual airlines within alliances with Imperx T9040.

Since the land coverage for an alliance depends on its individual members, we performed a detailed analysis for each airline within the alliance as well. The results are displayed in Figure 10. The figure depicts land coverage by each airline (monochrome bars), cumulative coverage when adding airlines together from left to right (line), as well as the contribution by each airline to the cumulative land coverage (red bars). The airlines are ordered in descending order by their individual land coverage. Thus, it should be noted that their contributions directly depended on this order, i.e., the first airline always had 100 percent contribution, while the second one having some overlaps with the first one would immediately have a contribution below 100 percent.

As seen in Figure 10c, the better coverage by Star Alliance could be explained by the dominant Turkish Airlines that alone covered above 30 percent of Europe, while for SkyTeam, three airlines had a dominant contribution, namely Air France, KLM, and Aeroflot, as depicted in Figure 10b. OneWorld in Figure 10a had a very logarithmic coverage contribution by its members, with British Airways still covering above 25 percent alone.

**Figure 11:**
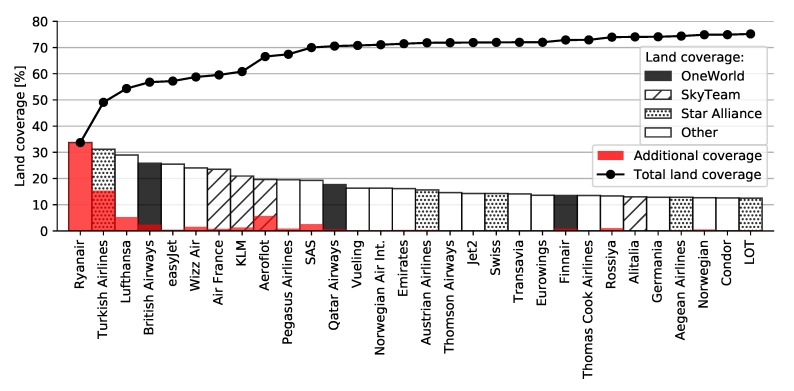
Cumulative land coverage by top 30 airlines with Imperx T9040.

We also performed land coverage analysis by individual airlines regardless of their alliance memberships, thus including those airlines that were not part of any alliance as well. Figure 11 shows that the largest land coverage by individual airline was achieved by Ryanair covering around 34 percent of Europe alone. Combined with Turkish Airlines from Star Alliance, they covered almost half of Europe. The other biggest contributors were Lufthansa and British Airways, covering central Europe, as well as airlines covering bordering areas such as Russian Aeroflot and Scandinavian SAS and Finnair.

**Figure 12:**
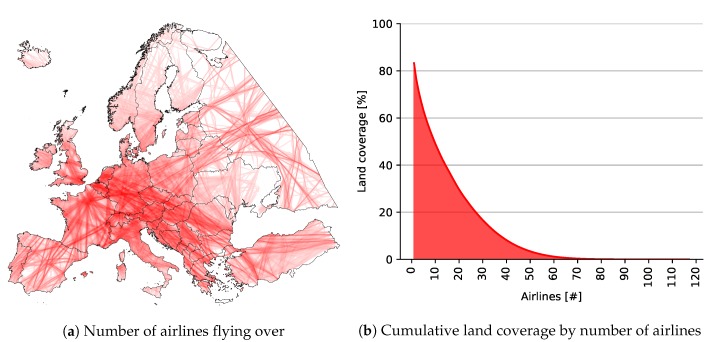
Land coverage by number of airlines flying over with Imperx T9040.

Finally, we depict a heat map in Figure 12 showing the land coverage by number of airlines, i.e., darker red color means that more airlines cover that area. It is evident from the figure that Central and southeast Europe were common routes for most of the airlines. The related graph depicts cumulative coverage by the number of airlines, showing that if at least 10 airlines were required to cover a certain location, the land coverage would be almost 50 percent. If at least one airline was required, the land coverage would be 83.28 percent, as previously calculated.

### 4.2. Temporal Resolution

**Figure 13:**
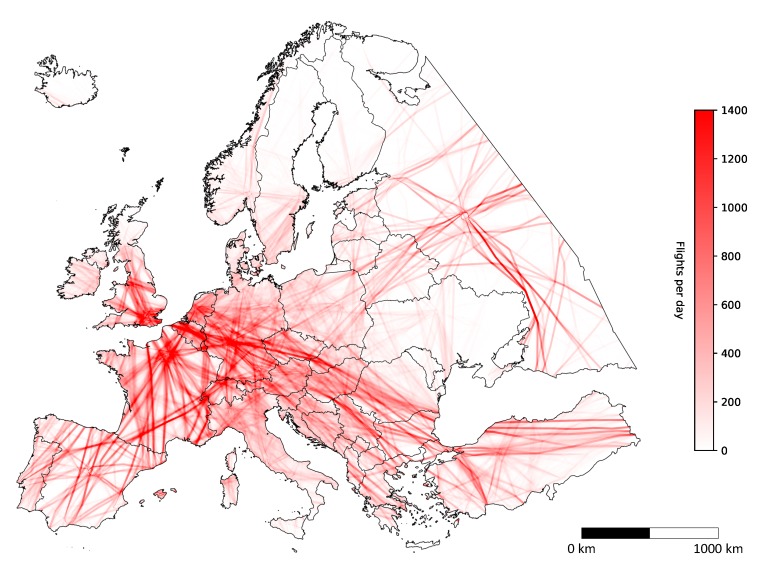
Number of flyovers by all fights with Imperx T9040.

**Figure 14:**
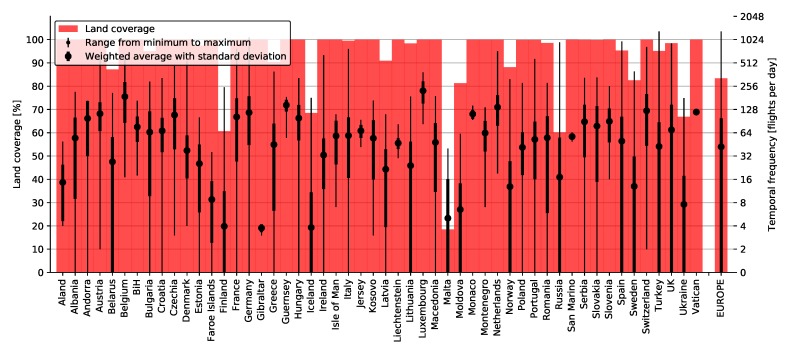
Temporal frequency (number of flyovers in a single day) for all flights using Imperx T9040.

Temporal resolution defines the time-frequency for taking aerial imagery, or more specifically for our case, the number of aircraft that fly over a certain area in a single day, i.e., 31 January 2018. Since the temporal resolution of a certain area directly depends on the land coverage, we continued our simulation only with Imperx T9040 as before, as all cameras gave similar coverage results, as seen in Figure 6. Figure 13 depicts the land coverage heat map, i.e., the number of flyovers in a single day, where the intensity of red color defines higher flyover frequency. Again, the results showed that 83.28 percent of Europe was covered by at least one flight per day. However, the results for temporal resolution also showed that over 60 percent was covered by at least 10 flights per day and almost 50 percent every hour on average. Most flyovers counted up to 1306 flights in a single day over a single point in Europe, while the average number of flyovers for the entirety of Europe was 42, i.e., if an image was taken on every flight, this would result in one aerial image every half an hour on average. A more detailed study was performed for each country in Europe, where the average number of flyovers in a single day was calculated, along with the maximum and minimum number of flyovers and their standard deviation. Results are depicted in Figure 14.

As seen from Figure 14, those 1306 flyovers previously calculated were located in Turkey. However, while having the highest number of flyovers, Turkey exhibited high deviation around its mean value of 43 flyovers. The second highest number of flyovers was exhibited by Germany with 1094 flyovers at maximum. However, there were 117 flyovers on average across the country with a considerably smaller deviation than in Turkey, i.e., the entire country was more evenly covered. Furthermore, the highest average number of flyovers was calculated for Luxembourg with 224 flyovers, which translated to one flyover every six minutes on average during a day. Other countries such as Monaco, San Marino, and Vatican also exhibited high coverage with a small deviation due to their small size and position.

**Figure 15:**
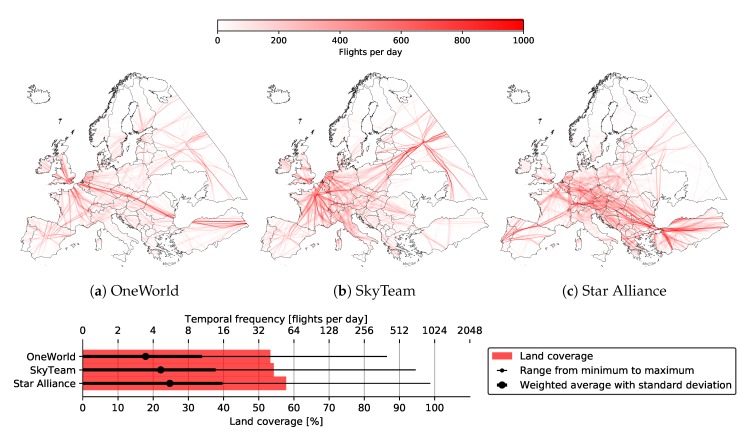
Temporal frequency for individual alliances with Imperx T9040.

A similar study was performed for individual alliances, namely OneWorld, SkyTeam, and Star Alliance for the entirety of Europe. Figure 15 depicts basic statistics for each alliance with 3.45, 4.65, and 5.56 average daily flyovers, respectively. The graph also shows that higher coverage and temporal frequency for alliances gave higher deviation as flights were more scattered around Europe. This is also visible in Figure 15a–c, depicting temporal heat maps for all three alliances, respectively.

### 4.3. Spatial Resolution

**Figure 16:**
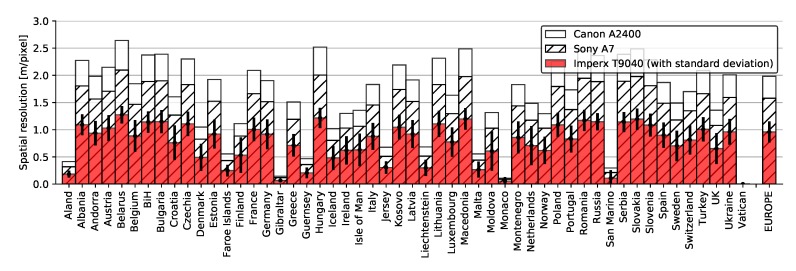
Spatial resolution expressed as GSD for individual countries/territories with the three cameras.

In this paper, we measured spatial resolution through GSD defined as an area size covered by a single pixel. This value depends directly on the altitude of an airborne platform, i.e., an aircraft, as well as the characteristics of the mounted camera. Consequently, in this simulation, we presented the results for all three used cameras. Figure 16 depicts GSD for all three cameras covering individual countries and territories in Europe, as well as the entirety of Europe.

**Figure 17:**
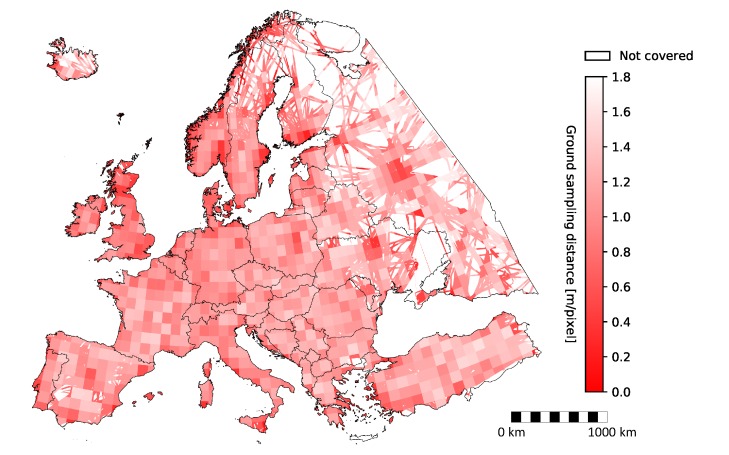
Spatial resolution expressed as average GSD for all flights with Imperx T9040.

The results showed that the average GSD for Europe was 0.96 meters per pixel with the Imperx T9040 camera, and only slightly worse for Sony A7 and Canon A2400, namely 1.58 and 1.98, respectively. The best average GSDs, namely below 0.5 meters per pixel, were achieved for countries/territories such as Vatican, Gibraltar, and Monaco due to their small size and vicinity to airports where aircraft fly at low altitudes. However, the worst average GSDs achieved for Belarus, Romania, and Hungary were still below three meters per pixel for all cameras. More detailed localization of average GSD across Europe for Imperx T9040 is shown in Figure 17.

Darker red color in Figure 17 depicts better average spatial resolution, while white color depicts no coverage at all. As seen from the figure, the best spatial resolution was achieved in the areas around airports, such as Moscow, Kyiv, Warsaw, Berlin, Barcelona, Madrid, and London. Less obvious airports were Frankfurt and Paris as these were overflown by a vast number of high-altitude flights, which consequently deteriorated the statistical value of the mean.

### 4.4. Storage Requirements

**Figure 18:**
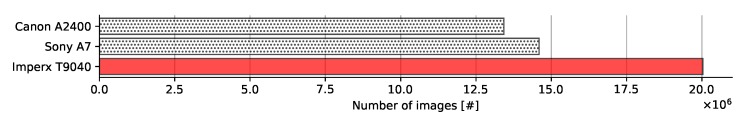
Number of acquired images in a single day for the entirety of Europe from all flights.

Deploying an airborne platform for remote sensing based on commercial flights requires a sufficient capacity for storing all acquired images. In this section, we estimate how much data were collected during a single day in the case when commercial flights were utilized as airborne platforms. We also accounted for 60 percent of image forward overlap, as suggested in [20], which generally increases the number of acquired images. The total number of images acquired for the entirety of Europe in a single day by all three cameras is depicted in Figure 18.

**Figure 19:**
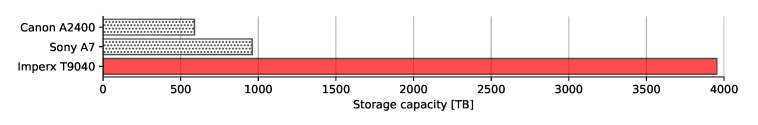
Storage required for storing images for the entirety of Europe in a single day from all flights.

Results in Figure 18 show that Imperx T9040 acquired over 20 million images in a single day, while Sony A7 and Canon A2400 acquired nearly 15 and 14 million images, respectively. While Imperx T9040 achieved wider horizontal coverage than other cameras (Figure 3), due to the smaller CCD height and consequently lower vertical FOV (Table 3), it achieved smaller vertical coverage along the flight trajectory. Consequently, it required more images to cover the same distance. Moreover, due to a larger image size (Table 3), it required more storage capacity as depicted in Figure 19. Namely, Imperx T9040 stored nearly four petabytes of aerial images in a single day, while Sony A7 and Canon A2400 stored almost one and 0.6 petabytes, respectively, due to the smaller image size and number of acquired images.

**Figure 20:**
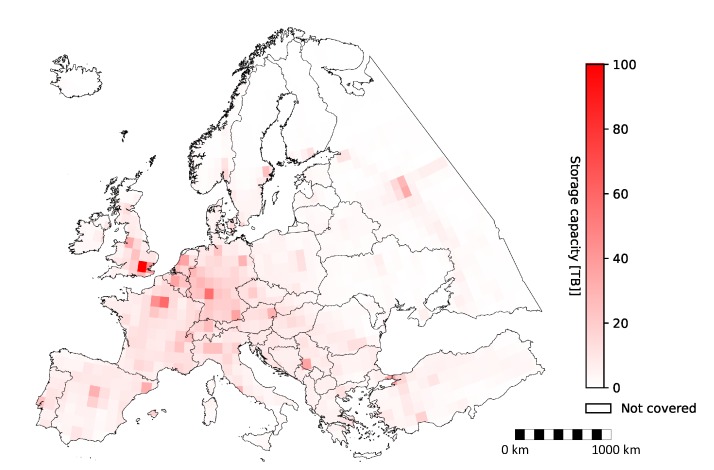
Storage required for storing aerial images taken from all flights with Imperx T9040.

Figure 20 depicts a heat map of the storage capacity required for storing all aerial imagery with Imperx T9040, where darker red means more data, while white means few or no data at all. The storage capacity shown in the figure is essentially a function of spatial and temporal resolutions depicted in Figure 13 and Figure 17, respectively. In other words, the storage size depends on the number of flights over a certain area and the altitude of those flights, i.e., lower altitudes result in a larger number of images.

**Figure 21:**
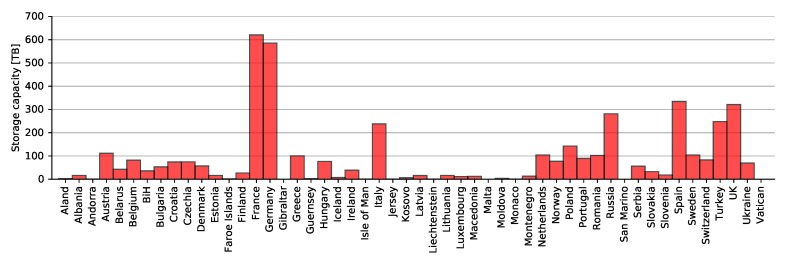
Storage required for storing images for individual countries/territories with Imperx T9040.

Consequently, the most storage capacity was required for the London area (approximately 100 TB), followed by Paris (approximately 59 TB) and Frankfurt (approximately 57 TB), which all represent major international airports. Note that these values were given only as examples for a single squared pixel closest to the referenced cities in Figure 20. More detailed values are given for separate countries/territories in Figure 21.

The results in Figure 21 show that the largest storage capacity was required for France, followed by Germany, Spain, the U.K., and Russia. As previously mentioned, storage capacity is a function of spatial and temporal resolutions and, in this case, the size of a country/territory as well. This explains why these countries were in the top five. Further analysis was done for three different alliances, as well as the top ten individual airlines when using Imperx T9040.

**Figure 22:**
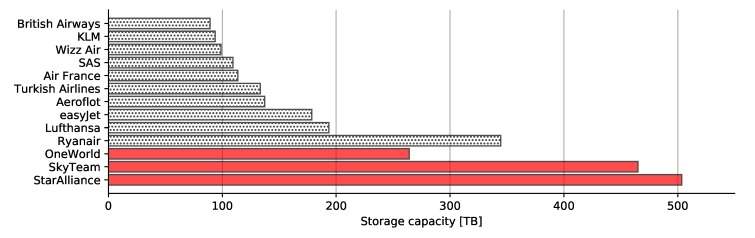
Storage required for storing images of the entirety of Europe by individual alliances and airlines.

Results in Figure 22 show that Star Alliance alone required over 500 terabytes of storage, which was one eighth of the entire capacity depicted in Figure 19. Star Alliance was followed by SkyTeam alliance and Ryanair, rather then OneWorld alliance. That said, Ryanair alone required almost 350 terabytes of storage capacity to store all data. Each aircraft regardless of the airline stored 126 gigabytes of data per flight on average, which included 625 images using Imperx T9040. It should be noted that this amount accounted only for images taken above land, i.e., excluding seas and oceans.

## 5. Discussion

The dataset used in this research included a single day for performing a simulation. Since commercial flights are planned according to a certain schedule, which is commonly defined on a daily basis for domestic and on a weekly basis for international flights, and are scheduled several months in advance with regards to seasonal demand [21], obviously different results would be obtained for different days in a week and for different seasons. However, these results still give valuable insights into the feasibility of using commercial flights as airborne platforms for remote sensing. Moreover, since the dataset used in this research came from the beginning of 2018, coverage had probably increased by 2020 due to an annual increase in air traffic by 1.9 percent [22].

**Figure 23:**
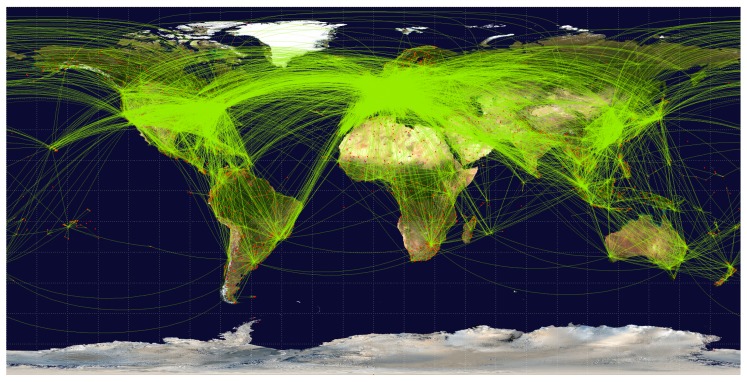
Global flight network (source: Wikimedia Commons, https://commons.wikimedia.org/wiki/File:World-airline-routemap-2009.png).

**Figure 24:**
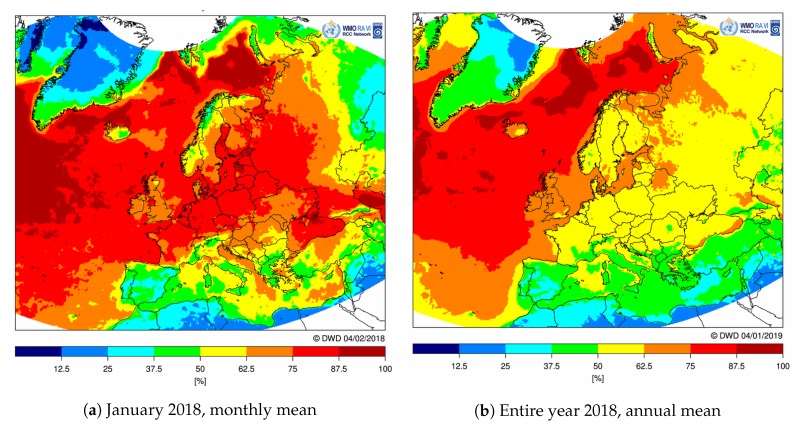
Cloud fractional cover (Source: Deutscher Wetterdienst, www.dwd.de).

Furthermore, since the used dataset included only Europe, a similar observation could be made by examining the spatial dispersion of the air traffic. According to IATA (International Air Transport Association: www.iata.org), Europe holds 26.3 percent of the entire world’s air traffic, while the Asia Pacific and North America hold 34.3 and 22.4 percent, respectively. Due to the larger size of the two continents, expected coverage and acquired average resolutions may be lower than for Europe. However, as reported by ICAO (International Civil Aviation Organization, UN specialized agency: www.icao.int) [23] and also seen in Figure 23, flights are unevenly dispersed across the two continents, and thus, the east coast of the U.S., Japan, and North China may achieve even better results than Europe. Finally, Africa, the Middle East, and Latin America exhibit significantly scarcer air traffic, comprising only 2.2, 10.4, and 4.5 percent of the world’s air traffic, respectively. Another aspect of aerial imagery that has to be taken into an account is the presence of clouds and Sun patterns, e.g., day and night. While Sun patterns are predictable and can be accounted for, clouds have an unpredictable behavior and significantly diminish a number of usable aerial images in the visible spectrum [24]. According to [25], containing data for average cloud cover in January 2018 (which is the same month as in the dataset used in this paper), most of Europe exhibits at least 62.5 percent of cloud cover, with the exception of the Mediterranean coast. Moreover, there are also daily variations of cloud cover, namely during summer months, there is a clear peak in the afternoon hours and a minimum at nighttime, while winter months in Europe show no significant diurnal variability [25]. Annual cloud cover is somewhat better, as seen in [25] (Figure 24b); however, most of Europe still exhibits 50 percent of cloud cover.

The limitations of aerial imagery due to the fact that Earth is on average 63 percent cloud-covered each day [26] is a challenge for other airborne platforms, as well, such as satellite platforms and high-altitude aircraft used for aerial imagery [27]. This issue has been tackled by different studies in the context of cloud cover estimation [25,28], cloud removal [29,30], as well as optimization of flight schedules [26]. Similar approaches can be applied in the context of this paper as well; however, a more comprehensive study on this subject is out of the scope of this paper.

The challenges described above are somewhat limited to the visible spectrum, which was the main focus of this paper. However, the results of this study could be applied for other spectra, as well. For instance, commercial aircraft can be equipped with Synthetic Aperture Radar (SAR) or with near-infrared (NIR) cameras that can (partly) penetrate clouds [31] and thus mitigate the cloud limitations. That said, going beyond remote sensing in the form of aerial imagery, usage of commercial flights could be extended to different types of sensing, as well, such as meteorological parameters, as proposed in [32]. The results obtained in this study could also be used to provide some insights into the the usability of commercial flights for those scenarios, as well.

Finally, in order to realize fully the scheme proposed in this paper, one would require a complete system design by adapting aircraft with mounted cameras, installing onboard storage, securing the uplink to cloud storage at airports, implementing image transformation algorithms that can sync geometric and spectral characteristics of acquired images, as well as solving the organizational, standardization, legal, and financial challenges of coordinating different airlines, assuring that all partners follow the same standards, and so forth. However, answering these questions is out of the scope of this paper, as the goal of this paper is to give insights into the benefits of the proposed scheme, i.e., to show if all the above is even worth it. We believe that the results are highly encouraging and that this approach is worth pursuing forward with additional simulations and analysis.

## 6. Conclusion and Future Work

In this work, we performed a simulation and a detailed analysis of applicability when using commercial flights as an airborne platform for remote sensing in the form of aerial imagery. The results showed the realistic feasibility of such a scheme with 83.28 percent of Europe covered. Such high coverage, along with the ground sampling distance of 0.96 meters per pixel and the temporal frequency of one image every half an hour provides benefits in comparison with satellite and drone imagery or their combination. Furthermore, an average size of 126 gigabytes of images acquired during a single flight certainly seems manageable in terms of storage and applicable for practical implementation. The data could be downloaded after each landing and uploaded to the cloud. Finally, the results showed that it was not necessary to include all airlines in order to achieve high coverage and frequency, which makes this scheme even more feasible.

For future work, our goal is to obtain even more realistic coverage with aerial imagery by including cloud coverage at different altitudes, as well as Sun patterns, e.g., day/night, time of day, cloud shadows, etc. Furthermore, depending on the available data, we aim at performing a worldwide simulation when using commercial flights as airborne platforms. This includes seas and oceans, as well, which are important for search and rescue operations, as well as environmental science. Finally, we plan to perform a more detailed analysis by taking into account the type of aircraft, which plays an important role when adapting commercial aircraft for airborne photography.

## Figures and Tables

**Table 1:** Metadata of the dataset segment containing the flight data. ICAO, International Civil Aviation Organization.

Data Field	Description	Example
Flight ID	Unique identifier for the flight	246321073
Aircraft ID	24 bit mode-S identifier in hexadecimals	3754508
Registration	Aircraft registration matched from the aircraft address	FGSQM
Equipment	ICAO aircraft designator, mapped from the address	B77W
Call sign	Up to 8 characters as sent from the aircraft transponder	AFR995
Flight number	Commercial flight number, interpreted from the call sign	AF995
ICAO	Airline three letter ICAO identifier extracted from the call sign	AFR
Airline	Name of an airline mapped with ICAO	Air France
Alliance	Alliance to which the airline belongs	SkyTeam

**Table 2:** Metadata of the dataset segment containing the trajectory data.

Data Field	Description	Example
Snapshot ID	Time of position update in seconds since 1 January 1970 00:00:00 UTC	1 504 228 476
Altitude	Height above sea level, in feet	10,972.80
Latitude	Floating point format	36.48667
Longitude	Floating point format	6.57222
Speed	Ground speed in knots	838.96

**Table 3:** Characteristics of the cameras used in the simulation.

Camera	Focal Length (mm)	CCD Width (mm)	CCD Height (mm)	Horizontal Pixels (#)	Vertical Pixels (#)	Image Size (MB)	FOV Horizontal (m)	FOV Vertical (m)
Canon A2400	5	6.17	4.55	4608	3 456	46	63.35	48.93
Sony A7	28	35.8	23.9	6000	4 000	69	65.18	46.22
Imperx T9040	35	47	22	10,440	4 800	207	67.76	34.89

**Table 4:** Countries and territories covered by the dataset.

Aland	Finland	Latvia	Romania
Albania	France	Liechtenstein	Russia
Andorra	Germany	Lithuania	San Marino
Austria	Gibraltar	Luxembourg	Serbia
Belarus	Greece	Macedonia	Slovakia
Belgium	Guernsey	Malta	Slovenia
BiH	Hungary	Moldova	Spain
Bulgaria	Iceland	Monaco	Sweden
Croatia	Ireland	Montenegro	Switzerland
Czechia	Isle of Man	Netherlands	Turkey
Denmark	Italy	Norway	Ukraine
Estonia	Jersey	Poland	U.K.
Faroe Islands	Kosovo	Portugal	Vatican

**Table 5:** Alliance statistics from the used dataset.

Alliance	Flights	Airlines ${}^{1}$	Aircraft ${}^{2}$	Distance (km)	Points	Altitude (km) **	Speed (km/h) **
OneWorld	2216	14/14	670/3 186	3,254,869	496,162	9.74	815.19
SkyTeam	3808	18/19	957/3 990	4,458,307	835,335	9.09	787.45
Star Alliance	4096	22/24	933/3 142	4,959,337	905,098	9.26	787.93
*Other*	22,339	309	4 832	26,453,450	4,771,206	9.22	777.44
**TOTAL**	**32,459**	**363**	**7392**	**39,125,963**	**7,007,801**	**9.25**	**783.05**

${}^{1}$ Total number of airlines in the alliance (source: official websites); ${}^{2}$ Total number of aircraft in the alliance (source: www.flightradar24.com); ** Values are calculated as the weighted averages based on a traveled distance.

## Abbreviations

The following abbreviations are used in this manuscript:

ETRS	European Terrestrial Reference System
LAEA	Lambert azimuthal equal area
CRS	Coordinate reference system
EPSG	European Petroleum Survey Group
IATA	International Air Transport Association
ICAO	International Civil Aviation Organization
DWD	Deutscher Wetterdienst
UAV	Unmanned aerial vehicle
UAS	Unmanned aerial system
UFO	Unidentified flying object
FOV	Field of view
AOV	Angle of view
CCD	Charged coupled device
GSD	Ground sampling distance
GIS	Geographic Information System
LiDAR	Laser imaging detection and ranging
SAR	Synthetic Aperture Radar
NIR	Near-infrared
3D	Three-dimensional
TB	Terabyte
WW-I	World War I
WW-II	World War II
CAGR	Compound Annual Growth Rate
USD	United States Dollar
